# Mutualism reduces the severity of gene disruptions in predictable ways across microbial communities

**DOI:** 10.1038/s41396-023-01534-6

**Published:** 2023-10-21

**Authors:** Jonathan N. V. Martinson, Jeremy M. Chacón, Brian A. Smith, Alex R. Villarreal, Ryan C. Hunter, William R. Harcombe

**Affiliations:** 1 Department of Ecology, Evolution, and Behavior, University of Minnesota, St. Paul, MN USA; 2 BioTechnology Institute, University of Minnesota, St. Paul, MN USA; 3 Department of Microbiology & Immunology, University of Minnesota, Minneapolis, MN USA; 4 Present Address: Minnesota Super Computing Institute, University of Minnesota, Minneapolis, MN USA

**Keywords:** Microbial ecology, Population genetics, Microbial ecology

## Abstract

Predicting evolution in microbial communities is critical for problems from human health to global nutrient cycling. Understanding how species interactions impact the distribution of fitness effects for a focal population would enhance our ability to predict evolution. Specifically, does the type of ecological interaction, such as mutualism or competition, change the average effect of a mutation (i.e., the mean of the distribution of fitness effects)? Furthermore, how often does increasing community complexity alter the impact of species interactions on mutant fitness? To address these questions, we created a transposon mutant library in *Salmonella enterica* and measured the fitness of loss of function mutations in 3,550 genes when grown alone versus competitive co-culture or mutualistic co-culture with *Escherichia coli* and *Methylorubrum extorquens*. We found that mutualism reduces the average impact of mutations, while competition had no effect. Additionally, mutant fitness in the 3-species communities can be predicted by averaging the fitness in each 2-species community. Finally, we discovered that in the mutualism *S. enterica* obtained vitamins and more amino acids than previously known. Our results suggest that species interactions can predictably impact fitness effect distributions, in turn suggesting that evolution may ultimately be predictable in multi-species communities.

## Introduction

Predicting evolutionary dynamics in microbial communities is critical for everything from understanding how pathogens in the human microbiome will respond to antibiotic treatment [1] to understanding how microbial contributions to global nutrient cycles will be impacted by climate change [2]. To understand and predict evolutionary outcomes in microbial communities, it is critical that we gain an understanding of how species interactions impact the average effect of mutations and the specific genes under selection, as well as how the effects of mutations are impacted as community complexity increases.

One way to assess the impact of species interactions on evolution is to evaluate impacts on the distribution of fitness effects caused by mutations in different conditions [3, 4]. By measuring the fitness of many mutants one can generate a distribution of fitness effects for a population in a defined environment [3, 4]. The mean of this distribution is a measure of how sensitive fitness is to perturbation by mutation for a given population in a defined environment [5, 6]. A more mutationally sensitive, or less robust, population will tend to have a more negative mean fitness score as most mutations that cause effects are expected to be deleterious [5, 6]. One approach that can be used to measure the distribution of fitness effects is transposon sequencing (or TnSeq) [7]. In this method, transposon mutant libraries are generated that contain thousands of mutants, each harboring a transposon inserted in a distinct location in the genome [8, 9]. Through sequencing the transposon library before and after growth under defined conditions, one can determine the change in mutant frequency and thereby the relative fitness of each insertion in discrete growth environments. While TnSeq studies often focus just on outliers to identify essential genes in different environments [8], the technique can also determine impacts on the entire distribution of fitness effects for a given species [4, 5, 7, 10, 11]. Indeed, the effect of mutations on a given strain is often observed to change in different environments [12–14].

Recent studies have shown that species interactions can change the fitness effects of mutations in a variety of ways, but the predictability of pair-wise effects remains unclear. For instance, a TnSeq study of *Escherichia coli* found that knockouts of several vitamin and nucleotide biosynthesis genes were deadly in monoculture, but viable in the presence of a phototrophic bacterium [10]. Another study tested the same *E. coli* library with different species of bacteria and fungi, and found that interactions with other species can result in either alleviation or exacerbation of fitness costs caused by transposon insertion [11, 15]. Similarly, co-infection has been shown to either increase or decrease the number of essential genes in the pathogen *Aggregatibacter actinomycetemcomitans* depending on the co-infecting partner [16]. These studies demonstrate that fitness effects of species interactions can be idiosyncratic, however there may be some generalities based on how species interact. Specifically, competition may tend to render gene disruptions more deleterious by further penalizing slow growth, while mutualism may tend to buffer the effect of gene loss through constraining growth rates to match those of a partner. To evaluate this possibility, data are needed on mutational effects in a system in which species identity can be held constant, while species interactions can be changed.

Another open question is whether the impact of mutations in complex communities can be predicted from the impact of mutations in simpler systems. Previous research found that only 28% of the interaction-associated fitness effects in a 4-species community were also observed in pairwise interactions [15]. This work suggests that higher order interactions (i.e., emergent properties) are prevalent and that the impact of mutations in communities may be difficult to predict from pairwise interactions. However, this contrasts with many studies suggesting that higher order interactions are relatively rare in microbial communities [17, 18]. The predictability of mutational impacts in a community setting remains unclear.

**Fig. 1:**
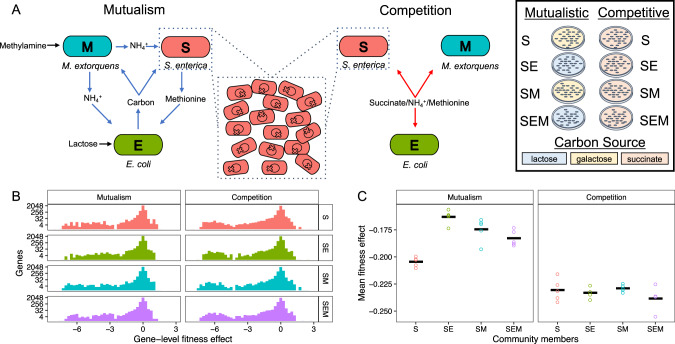
Distributions of fitness effects during mutualism and competition. **A** Interactions between *S. enterica* (S), *E. coli* (E), and *M. extorquens* (M) can be switched from mutualism (left) to competition (right) by changing the media. Central dotted inset–*S. enterica* population is composed of a transposon insertion (gene knockout) library. The circle within each cell represents a DNA chromosome–each ‘X’ represents a transposon insertion site (knockout). The library was tested in monoculture, 2-species co-culture, and 3-species co-culture (rightmost box). Plate color indicates carbon source—yellow = galactose; blue = lactose; orange = succinate. **B** Histograms of gene-level fitness effects. The fitness effect of each gene is the average across five replicates within a given treatment. Fitness effect, as described in the Materials and methods, is the normalized fitness where a value of zero indicates a neutral knockout. The y-axis scaling is log_2_-transformed. **C** Means of the fitness effects over all genes within a replicate. Each point is the mean of one replicate’s fitness effects. The bold horizontal line is the mean of these means. The mean fitness effect of knockouts in S monoculture was significantly (*p* = 2e-10) negative. There was a significant increase in the mean fitness effect when S depended on E (*p* = 2e-7) or M (*p* = 1e-5). Dependence on both E and M did not additively increase mean fitness effect, and instead lowered the mean below that for dependence on either species (*p* = 1.8e-6). The mean fitness effect of knockouts in competitive treatments was significantly below zero (intercept *p* < 2e-16) and the community members had no effect (smallest *p* = 0.144).

Here we use a randomly barcoded transposon-mutant (RB-TnSeq) library of *Salmonella enterica* (abbreviated as “S”) to investigate how species interactions impact the average effect of mutations, and the effect of disrupting specific genes. Changes in the nutrients provided in the media were used to switch *S. enterica* between mutualism and competition with *E. coli* and *Methylorubrum extorquens* (abbreviated as “E” and “M”, respectively). Our strain of *S. enterica* secretes methionine [19]. In lactose minimal medium, this *S. enterica* strain forms an obligate mutualism with an *E. coli* methionine auxotroph by providing methionine in exchange for carbon byproducts (Fig. 1A) [20]. The *S. enterica* can also form an obligate mutualism with *M. extorquens* in galactose minimal medium providing carbon byproducts in exchange for nitrogen when methylamine is the only nitrogen source [21]. Thus, all three species form an obligate mutualism in lactose minimal medium with methylamine (Fig. 1A). However, if succinate, methionine, and ammonium are each provided in the medium, all three strains can grow independently and act as competitors of each other (Fig. 1A). We grew the *S. enterica* RB-TnSeq library alone, with *E. coli*, with *M. extorquens* and with both species in either mutualism or competition (Supplementary Fig. S1) and sequenced the transposon barcodes in a process called BarSeq [9]. Monocultures were grown in galactose minimal medium for comparison against mutualistic growth, and succinate minimal medium to compare against competitive growth. We compared the effect of co-culture relative to its paired monoculture to determine whether the distribution of fitness effects varies in competition versus mutualism. We ran all experiments on agar surfaces as spatial structure can increase selection for novel cooperation between cross-feeding partners [20, 22].

We found that species interactions changed both the average effect of disrupting a gene as well as which genes were under selection. When *S. enterica* engaged in mutualism it was less affected by gene deletions than in monoculture, while in the competitive environment species interactions had no significant impact on the average fitness effect of gene disruption. The BarSeq data highlighted some expected interaction-specific selection such as the increased selection on genes associated with nitrogen uptake when *S. enterica* was reliant on nitrogen from *M. extorquens* [10]. The BarSeq data also illuminated some unanticipated selection, such as changes in the importance of vitamin biosynthesis in mutualism. Our data suggest that selection in 3-species communities can be well predicted from fitness in pairwise associations.

## Materials and methods

### Bacterial strains

Strains used in this study (Supplementary Table S1) including *Salmonella enterica* LT2 (WH102), and *Methylorubrum extorquens* AM1 have been described in previous studies [20, 23]. Briefly, the *S. enterica* contains mutations in *metA* and *metJ* causing it to secrete methionine [19], and the *M. extorquens* has a deletion of *hprA* making it unable to assimilate carbon from methylamine. The *E. coli* strain was generated using a P1 transduction method [24, 25] to move the *metB* deletion from the Keio clone JW3910 into *E. coli* MG1655. The resistance cassette was removed with flippase [24, 25].

Single gene knockouts in *S. enterica* were constructed using P22 HT *int* transduction to move knockouts from the BEI Resources *S. enterica* 14028 s knockout library. BEI plate IDs for the strains used were: ∆*aceA* –SGD_156/157_Cm, NR-42890 well A02; ∆*panC* – SGD_051/052_Cm, NR-42877, well D10; ∆*ilvA* – SGD_156/157_Cm, NR-42890 well F08. Transductions were carried out in a similar fashion to the P1 transductions described above, however, antibiotic resistance cassettes were left intact.

### Media

Routine culturing of *E. coli* (E) and *S. enterica* (S) was carried out on Lysogeny broth (LB), while *M. extorquens* (M) was cultured on Nutrient broth. The BarSeq experiments were carried out on hypho minimal medium [26]. Each component (Supplementary Table S2) was sterilized before mixing. The carbon source of the mutualistic treatments was either 5.56 mM galactose (for S and SM) or 2.78 mM lactose (for SE and SEM). Competitive treatments were provided 8.33 mM succinate as the carbon source.

Mutualistic treatments without *M. extorquens* (S and SE) and the competitive treatments were provided 3.7 mM (NH_4_)_2_SO_4_ as the nitrogen source. Mutualistic treatments with *M. extorquens* (SM and SEM) were provided 3.78 mM Na_2_SO_4_ and 1.16 mM methylamine. The competitive medium was supplemented with 0.05 mM methionine to allow for unrestricted *E coli* growth. All media was supplemented with 1.2 µM ZnSO_4_, 1 µM MnCl_2_, 18 µM FeSO_4_, 2 µM (NH_4_)_6_Mo_7_O_24_, 1 µM CuSO_4_, 2 mM CoCl_2_, 0.33 µM Na_2_WO_4_, and 20 µM CaCl_2_.

### RB-TnSeq library construction for *Salmonella enterica*

A randomly barcoded transposon library (RB-TnSeq) was generated in *S. enterica* WH102 using the conjugation method previously described [9]. Briefly, the donor *E. coli* strain APA752 containing the suicide transposon-plasmid, pKMW3, was conjugated with WH102 at a 1:1 ratio on LB supplemented with 300 µM diaminopimelic acid for 24 h at 37 °C. Roughly 300,000 transconjugant colonies were scraped and resuspended in saline (0.9%). The cells were then diluted to OD_600_ 0.25 in LB with kanamycin (50 µg/mL) and grown to an OD_600_ of 0.97 prior to being frozen in 10% glycerol at −80 °C. The library was sequenced on a NovaSeq S1 (Illumina) 2 × 150 bp flow cell at the University of Minnesota Genomics Center and analyzed using the FEBA pipeline [9] against the *S. enterica* LT2 genome (GenBank accession: AE006468.2).

### BarSeq experimental setup

A 1 mL RB-TnSeq library aliquot was thawed, inoculated into 25 mL LB with 50 µg/mL kanamycin, and incubated at 37 °C until it reached mid-log phase (OD_600_ 0.64). *E. coli* (MG1655 ∆*metB*) was cultured overnight and then diluted to mid-log in LB 37 °C. *M. extorquens* ∆*hprA* AM1 was cultured for 2 days in nutrient broth at 30 °C. All species were washed and adjusted to OD_600_ = 0.2 (for *S. enterica* and *E. coli*) or 0.4 (for *M. extorquens)*. 25 µL of each species (~10^6^ cells) was plated onto minimal media (five replicates/treatment) then incubated at 30 °C.

To harvest cells from the experimental plates, 4 mL saline was pipetted into each replicate plate and colonies were scraped with a cell spreader. A sample of the cell slurry was plated for CFU quantification on hypho agar plates that were selective for each species (Supplementary Table S3). The remaining cell slurry was pelleted by centrifugation and frozen at −20 °C.

DNA was extracted from pelleted cells using the Qiagen DNeasy Blood and Tissue kit. The resultant DNA was quantified with the Quant-iT PicoGreen dsDNA Assay kit. We performed the BarSeq98 PCR method previously described [9] with NEB Q5 polymerase and 200 ng of genomic DNA. In total, 55 samples with unique sequencing index barcodes were sequenced on a single lane on a NextSeq P2 (Illumina) 1 × 100-bp run that generated 315 million reads.

### Data filtering

Data filtering followed much of the same procedure described previously [9]. First, we removed barcodes which were not in chromosomal genes. Second, we removed barcodes that fell in the first, or last, 10% of a gene. Third, we removed barcodes which had three or fewer reads in any Time 0 (T0) sample. All samples had a median of more than 50 reads per gene. Fourth, we removed genes from the analysis if any T0 sample had fewer than 30 reads total in a gene or fewer than 15 reads in the first or last half of the gene. This left 105,000 barcodes across 3550 genes. One sample failed to pass quality check scores (competitive SEM replicate 1) and was removed from the analysis.

### Barcode-level fitness calculation

We calculated barcode-level fitness using a hybrid of the approaches described previously [9, 27]. First, a pseudocount of 0.1 was added to each barcode count to avoid taking log of zeros. Second, we divided each replicate’s read counts by the average number of barcode reads in five reference genes which we expected to have no fitness effect when knocked-out; this was done per-sample (the reference genes were STM0604, STM1237, STM0329, STM2774, and STM0333). To put the normalized counts back onto the original count scale, we multiplied the within sample normalized counts by the average number of barcode reads in the five reference genes over all samples. Finally, barcode-level fitness was calculated by log_2_(normalized counts) − log_2_(normalized counts of T0 sample). There were five T0 samples. Each sample within each treatment was designated as replicate 1–5, and each used a different T0 sample for the fitness calculation. Barcode-level fitness variance was calculated as previously performed [9].

### Gene-level fitness calculation

Fitness for each gene within each replicate was calculated as a weighted average of barcode-level fitness calculations. Specifically, fitness was weighted by 1/barcode-level variance. We set the maximum weight a barcode could receive at 20 reads [9]. Gene-level fitness was then calculated as Σ(barcode fitnesses X barcode weights)/Σ(barcode weights).

We also corrected for chromosome position. Following a previously published protocol [9], within each sample, we calculated a rolling median of gene-level fitness along the chromosome with a window size of 251 genes. This rolling median was subtracted from each gene’s fitness to obtain the final gene-level fitness value used in downstream analyses.

### Statistics

We used the gene-level fitness calculations to determine the mean effect of mutations across all gene disruptions. We calculated the arithmetic average effect of all mutations for each replicate. To determine whether species presence had a significant effect on the mean of the fitness distributions we used a linear regression model with species presence coded as a binary variable (0 = absence, 1 = presence). The model assessed whether the average fitness differed significantly between the four treatments within each environmental condition. The model was run separately for the competitive and mutualistic environmental conditions.

To compare fitness values of specific gene deletions across species addition treatments we used the same linear regression approach as above, but applied per-gene. To correct for multiple comparisons, we controlled the false-discovery rate by adjusting the *p* values across genes using Benjamini-Hochberg (BH) corrections and designated adjusted *p* < 0.05 as significant.

To predict gene fitness in the three-species communities, three different methods were tested. The additive, strongest, or average effects of E and M terms for each gene were added to the monoculture fitness for each gene. Predictions for all genes were correlated with the observed ESM fitness value and the *R*^2^ was examined. To compare prediction metrics a bootstrap analysis with 2001 iterations (with resampling) was performed. For each iteration, prediction values and ESM values were resampled, *R*^2^ values were calculated, and the frequency of times *R*^2^ values differed for each prediction was calculated. This process was repeated independently for both competition and mutualism.

In the overrepresentation analysis (ORA), we filtered for significant terms (BH adjusted *p* < 0.05) and classified gene effects as either positive or negative. ORA was then performed separately on the genes classified as positive and negative using the “enrichKEGG” function from the R package, ‘ClusterProfiler’ (version 4.7.1, downloaded 3/1/23) [28] with a universe size of 3550.

### Growth measurements

Growth curves in liquid were performed on overnight cultures grown in minimal medium. Cells were washed three times in saline, adjusted to an OD_600_ of 0.2, and diluted 1:100 in the relevant minimal medium. Cells were incubated at 30 °C in a Tecan Infinite Pro200 and shaken at 432 rotations per minute. Growth curve parameters were obtained by fitting a Baranyi function to the OD_600_.

Growth measurements on solid agar were performed by growing bacteria as in the ‘BarSeq Experimental Setup’ except that wildtype *S. enterica* was used in place of the library (Supplementary Fig. S2) using a methodology similar to previous timelapse imaging methods [29]. Plates were scanned (600 dpi) once per hour on an Epson Perfection V600 scanner at 30 °C. Lawn density was calculated by converting to gray scale, performing a Gaussian blur, and measuring the mean gray value for each plate using a custom script written with assistance from GPT-4 [30]. Growth curve parameters were obtained by fitting a Baranyi function to the mean gray value [31].

### Spent media preparation

Spent media was prepared by inoculating hypho minimal media with a 1:100 dilution of washed cells grown overnight. *M. extorquens* spent media was prepared in SM competitive broth (Supplementary Table S2). *E. coli* spent media was prepared in hypho with lactose and methionine. *E. coli* were grown to mid-log phase (OD_600_ ~ 0.2) or stationary phase (OD_600_ ~ 0.4). Spent media was supplemented with 1% galactose, 10% P solution, and 10% S solution. *S. enterica* spent media was made in either galactose or succinate + methionine hypho. Spent media from mid-log phase was then mixed at a 1:1 ratio with fresh galactose hypho. All spent media was centrifuged and then filter sterilized (0.2 µm).

### Vitamin and amino acid gradients

Galactose or succinate + methionine hypho were supplemented with either calcium pantothenate (0.5 µM to 5e-7 µM; Fisher Scientific) or DL-isoleucine (1 mM to 1e-6 mM; Alfa Aesar). The ∆*panC* and ∆*ilvA* were grown overnight, washed, adjusted to OD_600_ = 0.2, and diluted 1:100 before growth was measured.

### High performance liquid chromatography (HPLC) analysis

Quantification of organic acids in spent media was completed using a Dionex UltiMate 3000 RS HPLC system equipped with an Acclaim organic acid (OA) 5 μm 120 A° 4.0 × 250 mm column and accompanying guard column. Analyte separation was achieved using a 32-min isocratic run method consisting of an 8-min equilibration step followed by a 24-min static flow step with 100 mM NaSO_4_ (pH adjusted to pH 2.6 using CH_3_SO_3_H) at a rate of 1 mL/min. The RS column oven compartment temperature was maintained at 30 °C and the RS diode array was configured to collect UV readings at a wavelength of 210 nm with default frequency. All standards and samples were filtered through 0.22 μm polyethersulfone polymer filter membranes prior to injection (6 μL per sample). Chromeleon software (v.7.0) was used to configure all run sequence settings as well as view chromatogram data. Analyte peaks were identified, gated, and measured via the integrated Cobra Wizard prior to data export and processing.

## Results

The overall goal of our study was to determine how ecological interactions (i.e., mutualism versus competition) impact the distribution of fitness effects of mutation during growth on agar plates. The BarSeq experiment resulted in 3550 *S. enterica* gene disruptions which passed quality control (see Methods) and therefore entered downstream analyses. The *S. enterica* populations from which the BarSeq data were obtained experienced a similar number of generations between treatments within each ecological category of mutualism or competition (Supplementary Fig. S3), which allowed us to make direct comparisons within a category but not between. In the mutualism treatments all co-cultures grew more slowly than the monoculture (Supplementary Fig. S2). *S. enterica* had slightly but significantly lower yield in competitive co-culture than monoculture consistent with weak competition (*t* test, *p* < 0.05, Supplementary Fig. S3).

### Mutualism alters the distribution of fitness effects

As expected, most gene disruptions were neutral, with a longer tail towards knockouts causing low fitness than high fitness in all treatments (Fig. 1B). The large number of neutral mutations caused the median to be close to zero in all cases (Supplementary Fig. S4). The mean effect of mutations was negative in all cases, as the majority of mutations that altered fitness were deleterious (Fig. 1C).

To specifically evaluate the impact of species interactions on the effect of mutations we performed linear regression analysis with the presence/absence of each species coded as a binary 1 or 0 (Supplementary Tables S4, S5). The mean fitness of gene deletions was negative in each monoculture, because of the strongly deleterious nature of some mutations (Fig. 1C, galactose monoculture intercept = −0.205 (*p* < 0.001), succinate + methionine monoculture intercept = −0.231 (*p* < 0.001)). Species composition significantly impacted the mean fitness effect of mutation in the mutualistic environment (*R*^2^ = 0.80, *p* < 0.0001, *F* = 26.43). The mean fitness effect of mutations was less deleterious when *S. enterica* depended on either *E. coli* (average increase in fitness of *β* = 0.04, *p* < 0.0001) or *M. extorquens* (*β* = 0.03, *p* < 0.0001) compared to when *S. enterica* was grown by itself (Fig. 1C, left). There was not a significant effect of species across competitive treatments (*R*^2^ = 0.01, *p* > 0.05, *F* = 1.09).

**Fig. 2:**
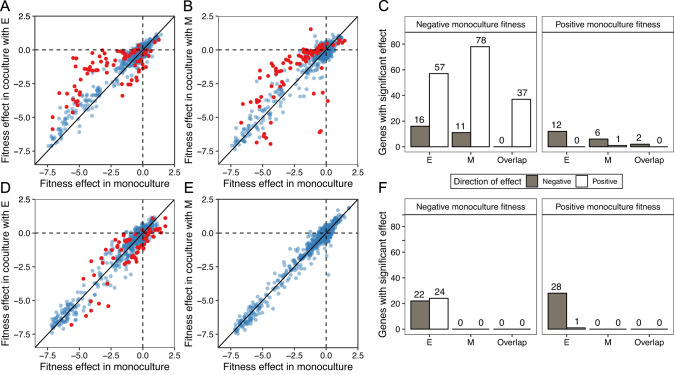
Mutualism tends to rescue mutants while competition slightly decreases mutant fitness. The mean fitness effect of each gene knockout in monoculture vs. co-culture with *E. coli* (**A**, **D**) or *M. extorquens* (**B**, **E**). The top row shows mutualistic co-cultures while the bottom row shows competitive co-cultures. Each point represents the mean fitness of one gene knockout across 5 replicates. Red dots indicate the co-culture fitness value differed significantly from the monoculture fitness value. **C**, **F** The number of genes with significant changes in fitness, categorized by their fitness effects in monoculture: negative (left facet) and positive (right facet). Gray bars indicate genes that exhibited significantly lower fitness in co-culture (with either E or M) compared to monoculture. In contrast, white bars denote genes that showed significantly higher fitness in co-culture. The overlap bars represent genes that experienced consistent directional changes in fitness in the presence of either E or M.

We investigated what drives the average effect of mutations to be less deleterious when *S. enterica* is engaged in mutualism. Fitness of knockouts in monoculture and mutualistic co-culture were generally similar (*R*^2^ = 0.87 for S vs. SE, 0.85 for S vs. SM, Fig. 2A, B). However, some knockouts had different fitness in co-culture than in monoculture. In mutualism, deleterious mutations tended to increase in fitness when *S. enterica* grown with either *E. coli* (binomial test, *p* < 0.001, Fig. 2C) or M. *extorquens* (binomial test, *p* < 0.001, Fig. 2C). There was a substantial degree of overlap in gene disruptions that were rescued by both *E. coli* and *M. extorquens* in mutualism (Fig. 2C). Conversely, mutations that were beneficial in monoculture were more likely to decrease in fitness when grown in mutualistic co-culture with *E. coli* (binomial test, *p* < 0.001, Fig. 2C) but not with *M. extorquens* (binomial test, *p* = 0.125). However, the number (and magnitude) of beneficial mutants in monoculture was much lower than the number (and magnitude) of deleterious mutants, leading the overall shift in mean fitness effect to tend towards neutrality when *S. enterica* depended on a partner. Competition caused fewer significant changes in mutant fitness than the mutualistic treatments. Competition with *E. coli* caused significantly more mutations that were beneficial in monoculture to decrease than increase in fitness (binomial test, *p* < 0.001, Fig. 2F). Competition with *M. extorquens* caused no significant changes in fitness.

### Effects of increasing community complexity are predictable

**Fig. 3:**
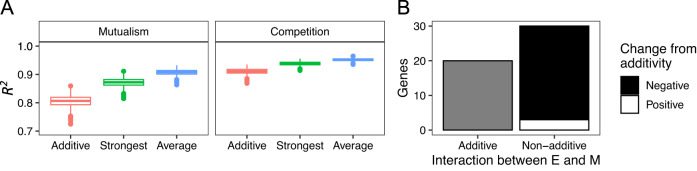
Predictability of fitness effects by community composition. **A** Distributions of 2001 bootstrapped *R*^2^ values for Additive, Strongest, and Average models in predicting mutant fitness in 3-species communities under mutualistic (left) and competitive (right) conditions. **B** The number of genes for which both E and M caused a significant main effect in mutualism, categorized by whether the interaction term was non-significant (additive—left) or significant (non-additive—right). The non-additive bar was colored by the number of genes in which the fitness was higher (positive—white) or lower (negative—black) than expected from additivity.

We tested whether fitness in the 3-species community could be predicted from the fitness in each 2-species community. We specifically evaluated whether fitness in the 3-species communities could be predicted from: (i) the additive effect on fitness of a knockout in each 2-species co-culture (ii) the strongest effect on fitness in either 2-species co-culture, or (iii) the average effect on fitness in each 2-species co-culture. All three prediction methods had high *R*^2^ (median > 0.8); however, the *R*^2^ was highest for predictions based on average fitness effects in both mutualism (Fig. 3A left) and competition (Fig. 3A right). The differences were slight particularly for competition, but a bootstrap analysis indicates a significant difference between predictive metrics for both mutualism and competition (bootstrap *p* < 0.05).

The fitness effects of knockouts in each co-culture tended to be sub-additive in the 3-species mutualism. When both *E. coli* and *M. extorquens* influenced the fitness of a knockout in their respective 2-species co-cultures, 30 out of the 50 knockouts had non-additive effects of adding both species (Fig. 3B). Of the 30 knockouts with non-additive effects, 27 exhibited sub-additivity: the typical result was that each co-culture species ameliorated the fitness cost of a knockout in comparison to monoculture, but the combined effect of the species in the 3-species co-culture was less than their sum.

### The fitness effects of mutualism were concentrated in specific pathways

**Fig. 4:**
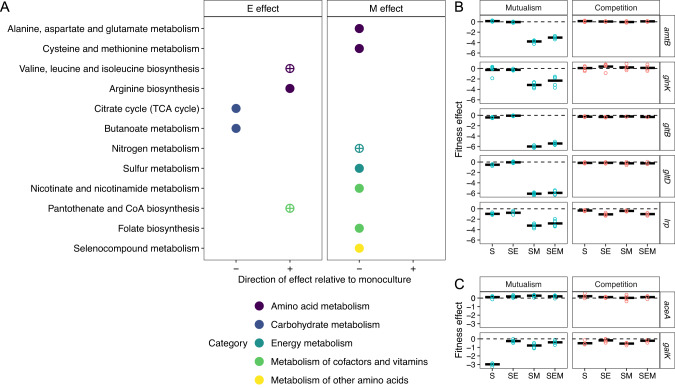
Overrepresentation analysis of genes with significant fitness effects. **A** Dotplot of KEGG pathways significantly overrepresented in the set of genes whose knockout fitness changed significantly as an effect of the presence of E or M (*p* < 0.05, BH multiple comparisons). Pathways overrepresented are indicated by circles. The plus/minus on the x-axis indicate the direction (relative to monoculture) of the fitness effect on the pathway. The color of the dots indicate the KEGG category of a pathway. Circles with crosses indicate pathways that were investigated further. **B** Normalized fitness scores for nitrogen metabolism genes in mutualistic (blue) and competitive (red) conditions. Each dot represents the average fitness in a replicate, and the bold horizontal line represents the mean fitness for each gene across replicates. **C** Normalized fitness scores in representative carbon metabolism genes for acetate (*aceA*) and galactose (*galK*) catabolism.

In addition to investigating how species interactions impacted the distribution of fitness effects, we also sought to investigate how species interactions altered the specific genes that contribute to fitness. We were particularly interested in understanding the mechanisms of interaction within the mutualism, so we performed ORA to identify pathways that become more and less important as a result of species interactions (Fig. 4A). We determined the effect of species composition on the fitness of each gene for each ecological treatment using multiple linear regression (Supplementary Fig. S5).

The presence of mutualists altered the relative importance of nitrogen and carbon metabolism. Reliance on ammonium from *M. extorquens* made knockouts highly deleterious for genes involved in nitrogen stress (*lrp, glnK*), ammonium transport (*amtB*) or glutamate biosynthesis (*gltB, gltD*). These knockouts were neutral in monoculture (Fig. 4B). Similarly, reliance on carbon from *E. coli* altered the importance of genes involved in carbon metabolism (Fig. 4A). However, genes for acetate and galactose metabolism were less important in the mutualisms than anticipated (Fig. 4C, Supplementary Fig. S6). This suggests that neither acetate nor galactose are the sole source of carbon that *S. enterica* acquires from *E. coli*. Future work will be required to identify the complete identity of carbon exchanged in this mutualism.

We found that several vitamin and amino acid biosynthesis pathways became less important in the presence of mutualistic partners suggesting the possibility of additional cross-fed metabolites (Fig. 4A). Based on this analysis, we further investigated the possibility that unexpected metabolites were also being provided from *E. coli* and *M. extorquens* to *S. enterica*.

### Vitamin and amino acids were also involved in cross-feeding

**Fig. 5:**
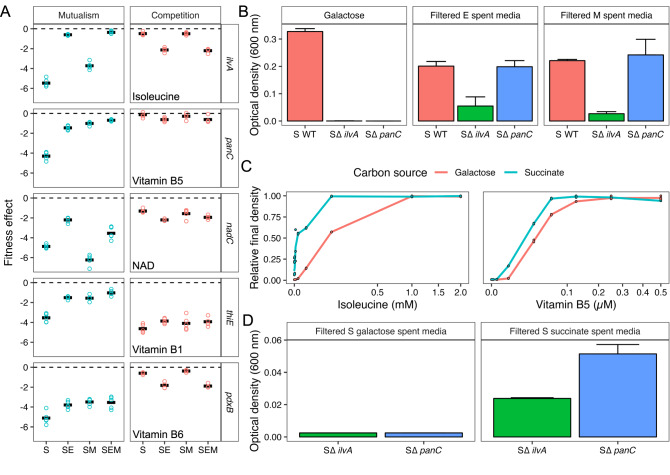
Effect of ecology on vitamin, cofactor, and amino acid metabolism gene fitness. **A** Normalized gene fitness scores for representative genes involved in biosynthesis. **B** Final OD_600_ of wild type and mutant *S. enterica* (S) cultivated in fresh galactose media (left), *E. coli* (E) spent media (middle), and *M. extorquens* (M) spent media (right). All experiments were performed in triplicate. **C** *S. enterica* ∆*ilvA* mutant (left) and ∆*panC* mutant (right) normalized yield on galactose and succinate minimal media with varying amounts of supplementation. Note that the y-axis units – relative final density – is the OD_600_ at a specified nutrient concentration supplemented into the medium divided by the maximum OD_600_ for each supplemented nutrient. The x-axis is square root transformed. All experiments were performed in triplicate. **D** Final OD_600_ of mutants ∆*ilvA* and ∆*panC S. enterica* grown in spent media prepared from wild type *S. enterica* grown to mid-log phase in galactose (left) or succinate (right) minimal media. All experiments were performed in triplicate. For all of the absorbances shown (**B**–**D**), the absorbance of the medium blanks were subtracted from the experimental treatments’ absorbance values.

Loss of biosynthetic genes for vitamins (B1, B5, B6), isoleucine and the co-factor NAD were less deleterious in mutualistic co-culture than monoculture on galactose (Fig. 5A). These results suggest that partner species may provide these nutrients to *S. enterica*. However, in our competitive environment when *S. enterica* was grown in monoculture on succinate we also saw that losing these genes had little impact on fitness. To further investigate these effects in vitro, we created new knockouts in *S. enterica* for critical genes in isoleucine (*ilvA*) and vitamin B5 (*panC*) biosynthesis.

Knockout constructs supported that mutualism altered the need for isoleucine and vitamin B5 biosynthesis. Neither ∆*panC* nor ∆*ilvA* could grow in isolation in galactose minimal medium (Fig. 5B); however, they could be rescued by either B5 or isoleucine (Fig. 5C). Additionally, each mutant could be at least partially rescued by growth in spent media from *E. coli* or *M. extorquens* (Fig. 5B right). This was also true for mutants of *nadC, pdxB*, and *thiE* from the BEI *S. enterica* knockout library (Supplementary Fig. S7). These data are consistent with *S. enterica* acquiring multiple metabolites from each mutualistic partner.

We investigated the neutrality of ∆*panC* and ∆*ilvA* mutants in our competitive monoculture treatment. We found that the ∆*panC* mutant was unable to grow in succinate. Interestingly, on succinate, the ∆*ilvA* mutant was only able to grow after ~60 h. Literature suggests that this growth is due to the gene *tdcB* [32], encoding an enzyme that yields 2-oxobutanoate (similar to *ilvA*) but only in the absence of sugar and oxygen. That said, over a 48-hour period, we found that neither mutant was capable of substantial growth on succinate. Furthermore, the mutants needed more isoleucine or vitamin B5 to maintain growth on galactose than on succinate (Fig. 5C). We also found that both mutants grew to higher yields in spent media from wildtype *S. enterica* grown on succinate than on galactose (Fig. 5D). These results suggest that the mutants could acquire needed metabolites from wildtype *S. enterica* (as well as from mutualistic partners), but that both the demand and secretion of the metabolites changed as a function of carbon source.

## Discussion

Using a transposon library in a defined microbial consortium, we found that ecological interactions have distinct effects on the impact of mutations in *S. enterica*. The average fitness of all mutants was closer to neutral when *S. enterica* was engaged in mutualism than when it was grown alone. However, this buffering was not observed when *S. enterica* was in competition with the same species. We also found that the impact of mutations in a three-species community could be well predicted from the average impact of the mutations in each two-species co-culture. Investigation of the impact of specific gene disruptions in the different ecological communities illuminated the mechanisms of species interactions. The transposon mutant fitness data highlighted that additional essential metabolites can be obtained from other cells, but both the supply and demand of these metabolites was impacted by the carbon environment.

Mutualism buffered the effect of mutations in our system. The mean fitness of mutants was closer to zero in mutualism than monoculture for all treatments despite the fact that the partner species and mechanisms of mutualism were distinct. Mutualism with either *E. coli* or *M. extorquens* caused significant increases in fitness of deleterious mutations and decreases in fitness for beneficial mutations, again supporting that mutualism tends to reduce the impact of mutations on *S. enterica*. The buffering of fitness effects in co-culture is likely driven in part by generic effects on growth rate, as *S. enterica* grows more slowly in all mutualisms than it does in monoculture (Supplementary Fig. S2). However, there was not a consistent effect of growth rate on the average effect of mutations, as within co-cultures there was no significant correlation between growth rate and mean fitness (Supplementary Fig. S8). We also directly demonstrated that both *E. coli* and *M. extorquens* rescue the growth of specific mutants through the release of metabolites rather than solely growth rate effects.

Mutualism and competition had distinct impacts on the average effect of mutations. Competition did not alter the average effect of mutations relative to monoculture. The lack of impact on average fitness could in part be explained by the strength of competition. In particular, *M. extorquens* is a relatively weak competitor, though it does significantly reduce the total biomass of *S. enterica* relative to monoculture (*t* test, *p* < 0.05). The species ratios are also distinct in mutualism and competitive treatments (Supplementary Fig. S3C). The competition results highlight that the ability of mutants to obtain essential metabolites from other species is strongly influenced by the environment. One cannot predict the fitness effects of losing biosynthetic pathways simply from species presence in a community – instead, it is critical to know how species are interacting.

Our results suggest that the impact of mutations in complex communities can be predicted from the impact of mutations in simpler co-cultures. The fitness effect of mutations in the 3-species community could be well predicted from fitness effects in each 2-species co-culture. Our results extend findings with an *E. coli* BarSeq library in co-culture with cheese rind microbes [15]. The previous work highlights that higher order interactions are common, and the fitness effects of mutations often change as community complexity increases. However, they find that for 16 knockouts which are consistently impacted by co-culture, the fitness effect of increasing community complexity is additive. We found that species effects in our system were typically sub-additive, but an additive model was still able to provide strong predictions of fitness in the 3-species community. The prediction could be further improved by calculating the average fitness effect over the two 2-species communities. The predictability of fitness effects with increasing community complexity will assuredly vary across species and environments, though it is reassuring that some similar patterns are emerging between highly distinct experimental systems. Our results suggest that in some communities, selection, and therefore evolutionary dynamics may be predictable with data from simpler systems.

BarSeq identified unknown metabolite exchange in our mutualism. Disruption of genes involved in vitamin, amino acid, and cofactor biosynthesis was less deleterious in mutualism than in monoculture. To better understand how mutualistic interactions improved fitness, we generated knockouts for two genes that had large fitness improvements in mutualism: *ilvA*—a gene involved in isoleucine biosynthesis, and *panC*—a gene involved in the production of vitamin B5 (pantothenate). While neither mutant grew in isolation on galactose minimal media, each grew in the presence of either *E. coli* or *M. extorquens*. This suggests that *E. coli* and *M. extorquens* excrete isoleucine and vitamin B5 (or precursors for these molecules). Several previous studies have documented rescue of auxotrophs in co-culture. In *E. coli* it has been shown that amino acids [33, 34], cofactors, and vitamins [35] can be obtained from other cells. Additionally, the phototrophic bacterium *Rhodopseudomonas palustris* has been shown to rescue *E. coli* with knockouts of purine, vitamin B6, and NAD biosynthesis [10]. Previous work suggested that overproduction of essential nutrients may be favored to avoid bottlenecks in metabolism [36]. In our system *E. coli* and *M. extorquens* each rescued a similar set of *S. enterica* auxotrophs supporting that there are a set of metabolites commonly released into environments by different taxa.

Carbon sources in our media impacted the ability of auxotrophs to be rescued by other cells. Many biosynthetic genes that were critical in monoculture on galactose were far less important when *S. enterica* was grown in monoculture on succinate. This led us to the hypothesis that mutants could obtain essential metabolites from other *S. enterica* cells, but that the carbon source changed the amount of metabolites released or the amount of metabolites required to rescue growth. Experiments with ∆*panC* and ∆*ilvA* mutants supported that growth on succinate led to both more excretion of essential metabolites by *S. enterica* and less metabolite being required for mutant growth. This result parallels previous observations that carbon source alters the prevalence of cross-feeding [37, 38]. These results highlight that carbon source strongly impacts cross-feeding of nutrients, and the degree to which cross-feeding buffers the impact of gene loss.

There are a number of caveats when considering the general applicability of our results regarding mutualism, competition, and the distribution of fitness effects. First, we only studied one form of mutation: gene disruption. We did not study basepair changes which may be more common drivers of adaptation. Though it is worth noting that a recent study demonstrated that distributions of fitness effects derived from transposon mutagenesis were predictive of evolutionary dynamics observed in *E. coli* [4]. Second, the carbon source varied across our treatments, and we demonstrated that carbon source was sufficient to alter the fitness effects of mutants. We found that mutualism had consistent impacts of reducing the average effect of mutations even though the carbon source changed between mutualistic treatments from galactose to the carbon that *S. enterica* obtains from *E. coli*. Third, our system has relatively few species, primarily metabolic interactions, and an initially homogeneous environment. The ability to predict fitness in communities from co-cultures will assuredly vary as community and environmental complexity increase.

The challenges of predicting evolution in communities has long been appreciated [39]. However, we observed that the fitness effects of mutations could be predicted to a degree in synthetic communities. All mutualisms we evaluated buffered the impact of gene loss, and indeed there was substantial overlap in the functions that were buffered by different mutualistic species. Furthermore, fitness in co-cultures was sufficient to predict fitness in more complex microbial communities. Our results suggest that synthetic communities of mutualists may often be more robust to genetic perturbations than synthetic communities of competitors. Additionally, our results suggest that study of pairwise interactions may ultimately allow us to understand, predict, and ultimately manage evolutionary dynamics even in complex natural communities.

### Supplementary information

Supplementary Material

## Data Availability

Statistics and figure generation were performed using R 4.2.1 and Python 3.9 using custom scripts available at https://github.com/JonMartinson/ecology_DFE. Raw transposon sequencing data are available in the NCBI BioProject PRJNA1008691.
