# Supplementary material for: Mutualism reduces the severity of gene disruptions in predictable ways across microbial communities
Source: ISME J. 2023 Oct 21;17(12):2270–8. doi: 10.1038/s41396-023-01534-6 (PMC10689784; doi:10.1038/s41396-023-01534-6)
Supplement: Supplementary file 1 — Supplementary Material [file 41396_2023_1534_MOESM1_ESM.pdf]

Alias	Strain ancestor	Antibiotic Marker	Abx conc. (μg/mL)	Source	Relevant phenotype
S	*Salmonella enterica* LT2 metA* metJ*	NA	NA	Harcombe 2010	Methionine hypersecreter
E	*Escherichia coli* MG1655 ΔmetB	NA	NA	This study	Methionine auxotroph
M	*Methylorubrum extorquens* AM1 ΔhrpA	NA	NA	Marx 2008	Unable to assimilate carbon from C1 compouds
SΔaceA	*Salmonella enterica* LT2 metA* metJ*	Chloramphenicol	20	This study	Cannot use acetate as sole carbon source
SΔpanC	*Salmonella enterica* LT2 metA* metJ*	Kanamycin	50	This study	Vitamin B5 auxotroph
SΔilvA	*Salmonella enterica* LT2 metA* metJ*	Kanamycin	50	This study	Isoleucine auxotroph
BEIΔpdxB	*Salmonella enterica* 14028s	Kanamycin	50	Porwollik 2014	Vitamin B6 auxotroph
BEIΔnadC	*Salmonella enterica* 14028s	Kanamycin	50	Porwollik 2014	NAD auxotroph
BEIΔthiE	*Salmonella enterica* 14028s	Kanamycin	50	Porwollik 2014	Vitamin B1 auxotroph

Supplementary Table S1: Strains used for experiments. See materials and methods for description of mutant construction methods.

Type	Component	metric	S mutualism	SE mutualism	SM mutualism	SEM mutualism	S competitive	SE competitive	SM competitive	SEM competitive
	agar	%	1.5	1.5	1.5	1.5	1.5	1.5	1.5	1.5
C source	galactose	mM	5.56	0	5.56	0	0	0	0	0
	lactose	mM	0	2.78	0	2.78	0	0	0	0
	succinate	mM	0	0	0	0	8.33	8.33	8.33	8.33
auxotroph amendments	methylamine	mM	0	0	1.16	1.16	0	0	0	0
	methionine	mM	0	0	0	0	0.05	0.05	0.05	0.05
SO4 source	(NH4)2SO4	mM	3.7	3.7	0	0	3.7	3.7	3.7	3.7
	MgSO4	mM	0.814	0.814	0.814	0.814	0.814	0.814	0.814	0.814
	Na2SO4	mM	0	0	3.78	3.78	0	0	0	0
P source	K2HPO4	mM	14.5	14.5	14.5	14.5	14.5	14.5	14.5	14.5
	NaH2PO4	mM	16.3	16.3	16.3	16.3	16.3	16.3	16.3	16.3
Metals	ZnSO4	µM	1.2	1.2	1.2	1.2	1.2	1.2	1.2	1.2
	MnCl2	µM	1	1	1	1	1	1	1	1
	FeSO4	µM	18	18	18	18	18	18	18	18
	(NH4)6Mo7O24	µM	2	2	2	2	2	2	2	2
	CuSO4	µM	1	1	1	1	1	1	1	1
	CoCl2	µM	2	2	2	2	2	2	2	2
	Na2WO4	µM	0.33	0.33	0.33	0.33	0.33	0.33	0.33	0.33
	CaCl2	µM	20	20	20	20	20	20	20	20

Supplementary Table S2: Modified hypho media composition: Media composition for the mutualistic and competitive communities. Note that plate reader experiments were prepared with media that omits the agar.

Type	Component	metric	S	E	M
	agar	%	1.5	1.5	1.5
C source	galactose	mM	5.56	0	0
	lactose	mM	0	2.78	0
	succinate	mM	0	0	8.33
auxotroph amendment	methylamine	mM	0	0	1.16
	methionine	mM	0	0.05	0
SO4 source	(NH4)2SO4	mM	3.7	3.7	0
	MgSO4	mM	0.814	0.814	0.814
	Na2SO4	mM	0	0	3.78
P source	K2HPO4	mM	14.5	14.5	14.5
	NaH2PO4	mM	16.3	16.3	16.3
Metals	ZnSO4	µM	1.2	1.2	1.2
	MnCl2	µM	1	1	1
	FeSO4	µM	18	18	18
	(NH4)6Mo7O24	µM	2	2	2
	CuSO4	µM	1	1	1
	CoCl2	µM	2	2	2
	Na2WO4	µM	0.33	0.33	0.33
	CaCl2	µM	20	20	20

Supplementary Table S3: Species specific quantification agar composition.

ecology	term	estimate	std.error	statistic	p.value
mutualism	(Intercept)	-0.2045424	0.00340435	-60.082564	2.83E-20
mutualism	ETRUE	0.04143888	0.00481448	8.60712644	2.12E-07
mutualism	MTRUE	0.02996686	0.00481448	6.22431312	1.22E-05
mutualism	ETRUE:MTRUE	-0.0496794	0.00680871	-7.296453	1.79E-06
competition	(Intercept)	-0.2306512	0.0036842	-62.605462	1.47E-19
competition	ETRUE	-0.0025553	0.00521025	-0.4904426	0.63091732
competition	MTRUE	0.00159337	0.00521025	0.30581424	0.76394931
competition	ETRUE:MTRUE	-0.0068621	0.00759518	-0.9034797	0.38055223

Supplementary Table S4: Linear regression results for mutualistic and competitive communities.

ecology	r.squared	adj.r.squarec	sigma	statistic	p.value	df	logLik	AIC	BIC	deviance	df.residual	nobs
mutualism	0.83207469	0.8005887	0.00761237	26.4268193	1.95E-06	3	71.412283	-132.82457	-127.8459	9.27E-04	16	20
competition	0.17876241	0.01451489	0.00823813	1.08837206	0.38422952	3	66.4665202	-122.93304	-118.21085	0.001018	15	19

Supplementary Table S5: Linear regression model metrics.

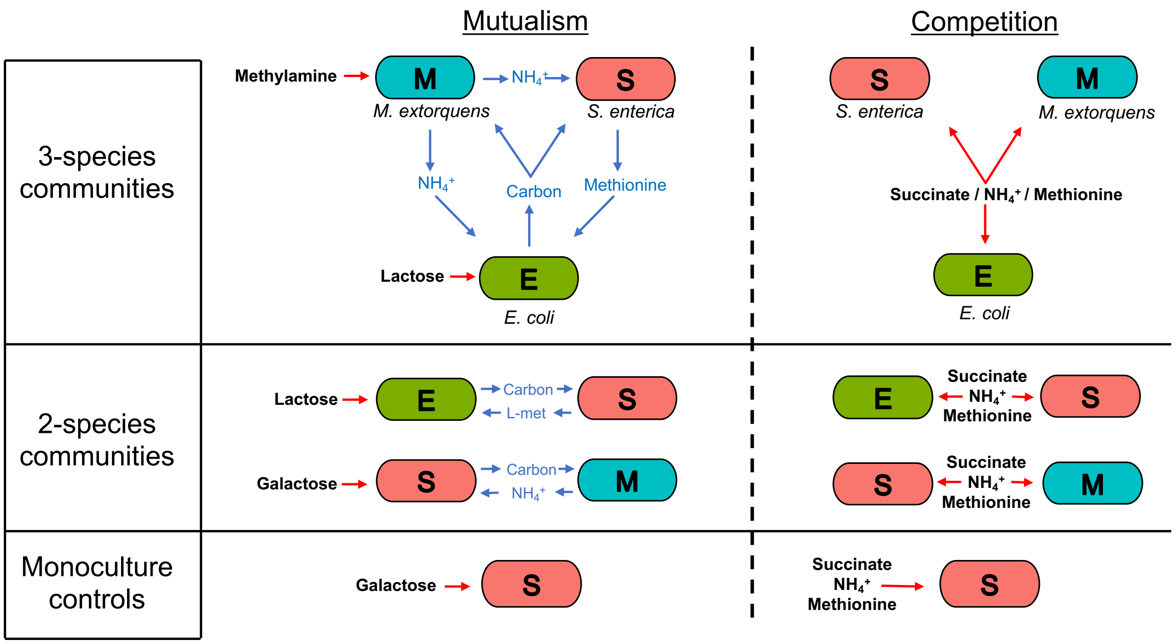

Supplementary Figure S1 – Schematic of species and media composition in experimental treatments. *S. enterica* (S), *E. coli* (E), and *M. extorquens* (M) were grown in mutualistic (left) and competitive conditions (right) in 3-species (top), 2-species (middle), and single-species treatments (bottom). Bold text and red arrows indicate a supplemented resource that is consumed by one or more species, while blue arrows designate the producer (shaft of arrow) and consumer (point of arrow) of a cross-fed resource.

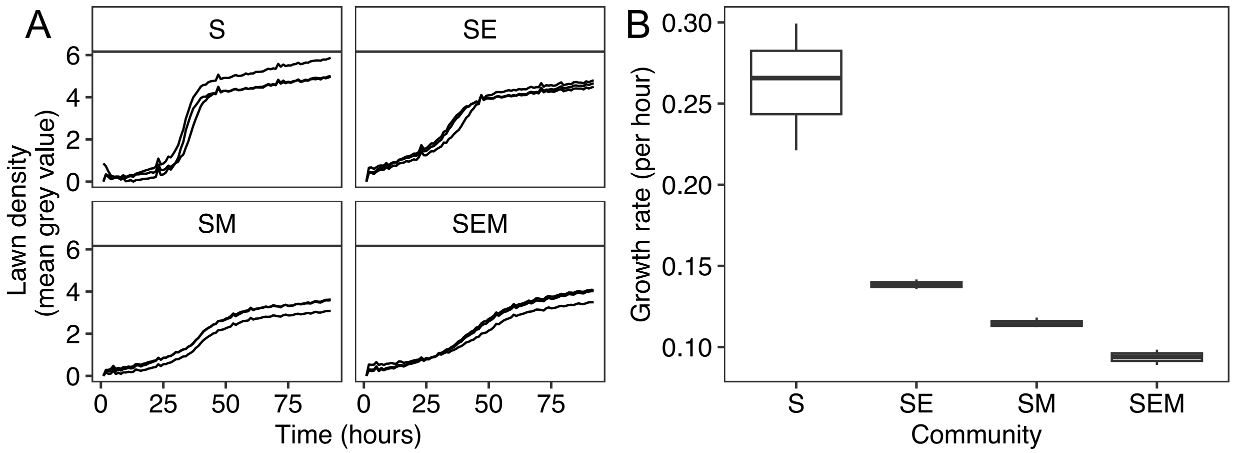

Supplementary Figure S2 – Growth on agar. Growth of communities measured by hourly automated scanning of each mutualistic community in triplicate. Density of bacterial lawns was approximated by converting each plate image to greyscale, then calculating the mean grey value through time **(A)**. Growth rates were fit using Baranyi curves **(B)** – S monoculture's growth rate is significantly greater than mutualistic co-culture (Tukey HSD, $p < 0.05$ for S vs SE, S vs SM, S vs SEM).

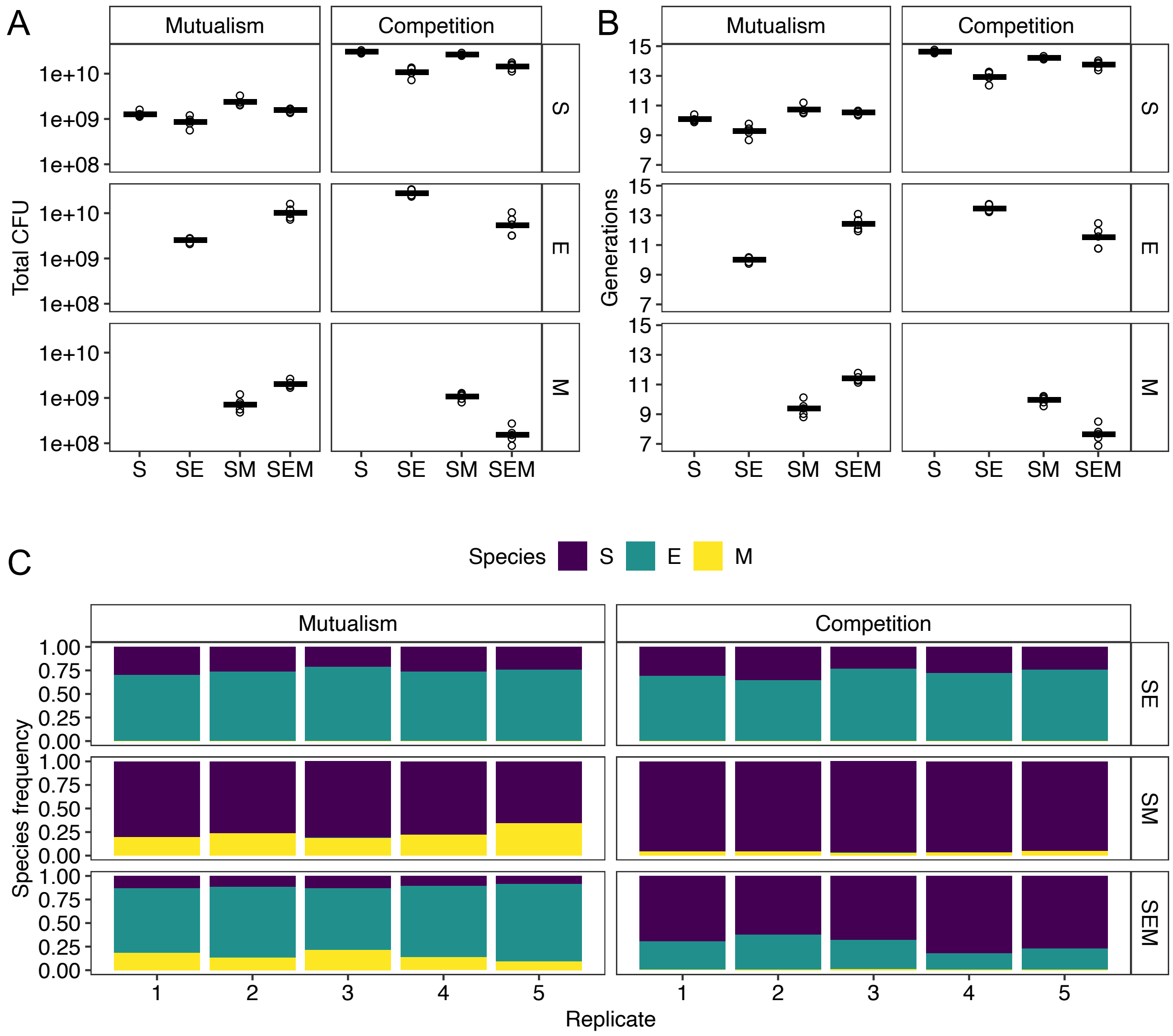

Supplementary Figure S3 – Species population sizes and generations. Total population size **(A)** and number of generations **(B)** for each species (indicated by y-axis faceting) for each replicate/treatment. **C** Species frequency for each co-culture replicate.

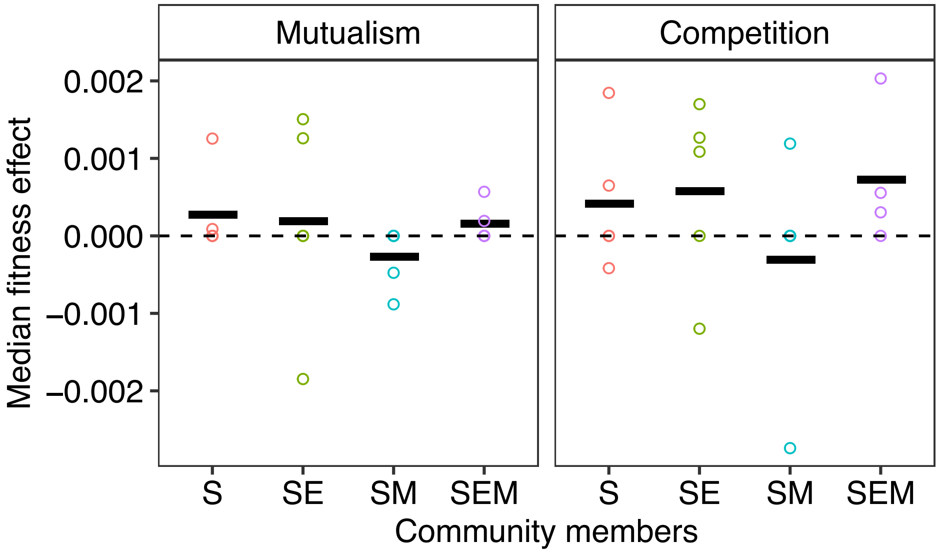

Supplementary Figure S4 – Median fitness effect is close to zero in all treatments. Median fitness effect over all genes within a replicate. Each point is the median value of one replicate's fitness effects. The bold horizontal line is the mean of these medians.

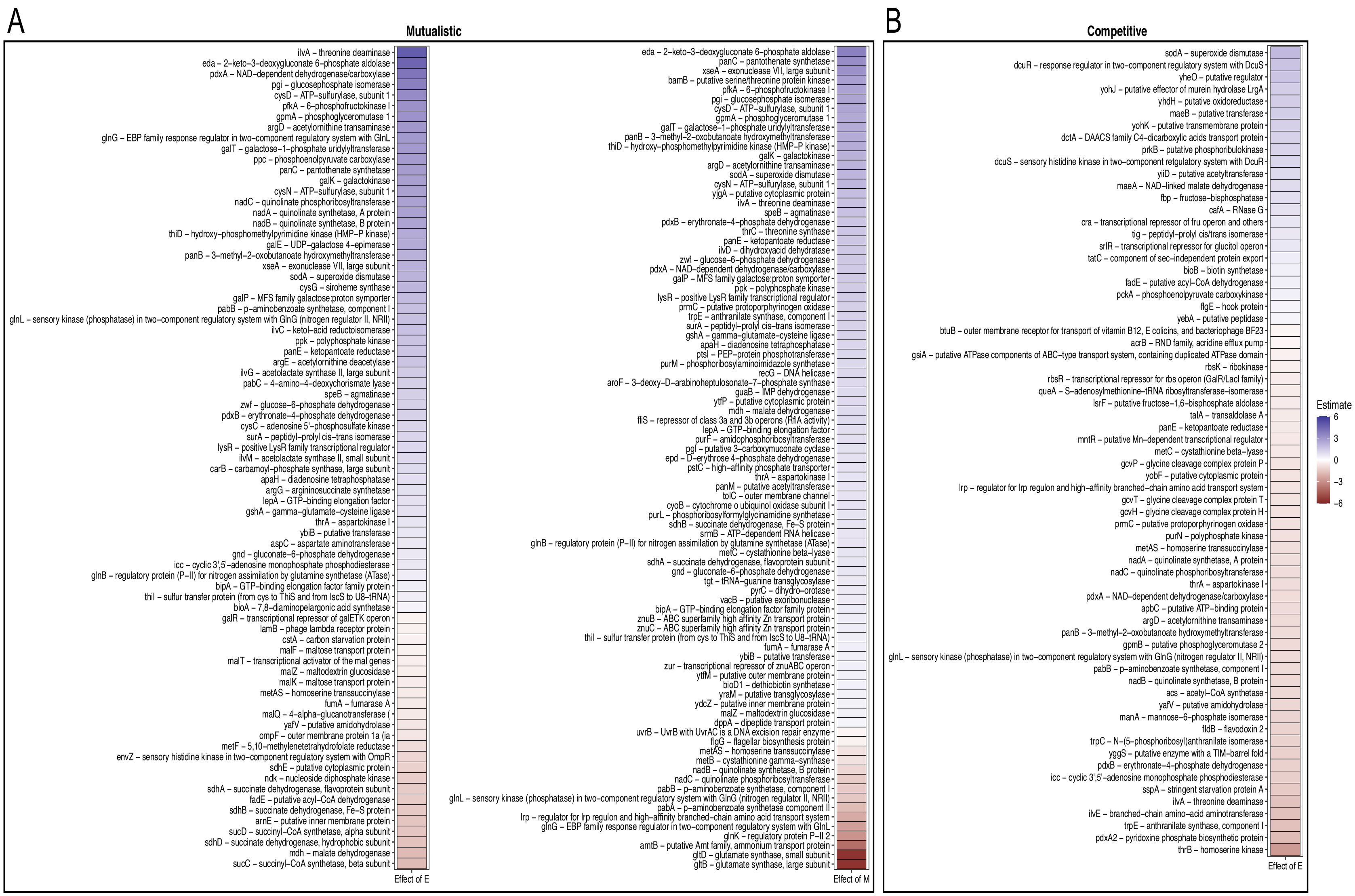
A
Mutualistic
ilvA – threonine deaminase
eda – 2–keto–3–deoxygluconate 6–phosphate aldolase
pdxA – NAD–dependent dehydrogenase/carboxylase
pgi – glucosephosphate isomerase
cysD – ATP–sulfurylase, subunit 1
pfkA – 6–phosphofructokinase I
gpmA – phosphoglyceromutase 1
argD – acetylornithine transaminase
glnG – EBP family response regulator in two–component regulatory system with GlnL
galT – galactose–1–phosphate uridylyltransferase
ppc – phosphoenolpyruvate carboxylase
panC – pantothenate synthetase
galK – galactokinase
cysN – ATP–sulfurylase, subunit 1
nadC – quinolinate phosphoribosyltransferase
nadA – quinolinate synthetase, A protein
nadB – quinolinate synthetase, B protein
thiD – hydroxy–phosphomethylpyrimidine kinase (HMP–P kinase)
galE – UDP–galactose 4–epimerase
panB – 3–methyl–2–oxobutanoate hydroxymethyltransferase
xseA – exonuclease VII, large subunit
sodA – superoxide dismutase
cysG – siroheme synthase
galP – MFS family galactose:proton symporter
pabB – p–aminobenzoate synthetase, component I
glnL – sensory kinase (phosphatase) in two–component regulatory system with GlnG (nitrogen regulator II, NRII)
ilvC – ketol–acid reductoisomerase
ppk – polyphosphate kinase
panE – ketopantoate reductase
argE – acetylornithine deacetylase
ilvG – acetolactate synthase II, large subunit
pabC – 4–amino–4–deoxychorismate lyase
speB – agmatinase
zwf – glucose–6–phosphate dehydrogenase
pdxB – erythronate–4–phosphate dehydrogenase
cysC – adenosine 5'–phosphosulfate kinase
surA – peptidyl–prolyl cis–trans isomerase
lysR – positive LysR family transcriptional regulator
ilvM – acetolactate synthase II, small subunit
carB – carbamoyl–phosphate synthase, large subunit
apaH – diadenosine tetraphosphatase
argG – argininosuccinate synthetase
lepA – GTP–binding elongation factor
gshA – gamma–glutamate–cysteine ligase
thrA – aspartokinase I
ybiB – putative transferase
aspC – aspartate aminotransferase
gnd – gluconate–6–phosphate dehydrogenase
icc – cyclic 3',5'–adenosine monophosphate phosphodiesterase
glnB – regulatory protein (P–II) for nitrogen assimilation by glutamine synthetase (ATase)
bipA – GTP–binding elongation factor family protein
thiI – sulfur transfer protein (from cys to ThiS and from IscS to U8–tRNA)
bioA – 7,8–diaminopelargonic acid synthetase
galR – transcriptional repressor of galETK operon
lamB – phage lambda receptor protein
cstA – carbon starvation protein
malF – maltose transport protein
malT – transcriptional activator of the mal genes
malZ – maltodextrin glucosidase
malK – maltose transport protein
metAS – homoserine transsuccinylase
fumA – fumarase A
malQ – 4–alpha–glucanotransferase (
yafV – putative amidohydrolase
ompF – outer membrane protein 1a (ia
metF – 5,10–methylenetetrahydrofolate reductase
envZ – sensory histidine kinase in two–component regulatory system with OmpR
sdhE – putative cytoplasmic protein
ndk – nucleoside diphosphate kinase
sdhA – succinate dehydrogenase, flavoprotein subunit
fadE – putative acyl–CoA dehydrogenase
sdhB – succinate dehydrogenase, Fe–S protein
arnE – putative inner membrane protein
sucD – succinyl–CoA synthetase, alpha subunit
sdhD – succinate dehydrogenase, hydrophobic subunit
mdh – malate dehydrogenase
sucC – succinyl–CoA synthetase, beta subunit
Effect of E
eda – 2–keto–3–deoxygluconate 6–phosphate aldolase
panC – pantothenate synthetase
xseA – exonuclease VII, large subunit
bamB – putative serine/threonine protein kinase
pfkA – 6–phosphofructokinase I
pgi – glucosephosphate isomerase
cysD – ATP–sulfurylase, subunit 1
gpmA – phosphoglyceromutase 1
galT – galactose–1–phosphate uridylyltransferase
panB – 3–methyl–2–oxobutanoate hydroxymethyltransferase
thiD – hydroxy–phosphomethylpyrimidine kinase (HMP–P kinase)
galK – galactokinase
argD – acetylornithine transaminase
sodA – superoxide dismutase
cysN – ATP–sulfurylase, subunit 1
yjgA – putative cytoplasmic protein
ilvA – threonine deaminase
speB – agmatinase
pdxB – erythronate–4–phosphate dehydrogenase
thrC – threonine synthase
panE – ketopantoate reductase
ilvD – dihydroxyacid dehydratase
zwf – glucose–6–phosphate dehydrogenase
pdxA – NAD–dependent dehydrogenase/carboxylase
galP – MFS family galactose:proton symporter
ppk – polyphosphate kinase
lysR – positive LysR family transcriptional regulator
prmC – putative protoporphyrinogen oxidase
trpE – anthranilate synthase, component I
surA – peptidyl–prolyl cis–trans isomerase
gshA – gamma–glutamate–cysteine ligase
apaH – diadenosine tetraphosphatase
ptsI – PEP–protein phosphotransferase
purM – phosphoribosylaminoimidazole synthetase
recG – DNA helicase
aroF – 3–deoxy–D–arabinoheptulosonate–7–phosphate synthase
guaB – IMP dehydrogenase
ytfP – putative cytoplasmic protein
mdh – malate dehydrogenase
fliS – repressor of class 3a and 3b operons (RflA activity)
lepA – GTP–binding elongation factor
purF – amidophosphoribosyltransferase
pgl – putative 3–carboxymuconate cyclase
epd – D–erythrose 4–phosphate dehydrogenase
pstC – high–affinity phosphate transporter
thrA – aspartokinase I
panM – putative acetyltransferase
tolC – outer membrane channel
cyoB – cytochrome o ubiquinol oxidase subunit I
purL – phosphoribosylformylglycinamidine synthetase
sdhB – succinate dehydrogenase, Fe–S protein
srmB – ATP–dependent RNA helicase
glnB – regulatory protein (P–II) for nitrogen assimilation by glutamine synthetase (ATase)
metC – cystathionine beta–lyase
sdhA – succinate dehydrogenase, flavoprotein subunit
gnd – gluconate–6–phosphate dehydrogenase
tgt – tRNA–guanine transglycosylase
pyrC – dihydro–orotase
vacB – putative exoribonuclease
bipA – GTP–binding elongation factor family protein
znuB – ABC superfamily high affinity Zn transport protein
znuC – ABC superfamily high affinity Zn transport protein
thiI – sulfur transfer protein (from cys to ThiS and from IscS to U8–tRNA)
fumA – fumarase A
ybiB – putative transferase
zur – transcriptional repressor of znuABC operon
ytfM – putative outer membrane protein
bioD1 – dethiobiotin synthetase
yraM – putative transglycosylase
ydcZ – putative inner membrane protein
malZ – maltodextrin glucosidase
dppA – dipeptide transport protein
uvrB – UvrB with UvrAC is a DNA excision repair enzyme
flgG – flagellar biosynthesis protein
metAS – homoserine transsuccinylase
metB – cystathionine gamma–synthase
nadB – quinolinate synthetase, B protein
nadC – quinolinate phosphoribosyltransferase
pabB – p–aminobenzoate synthetase, component I
glnL – sensory kinase (phosphatase) in two–component regulatory system with GlnG (nitrogen regulator II, NRII)
pabA – p–aminobenzoate synthetase component II
lrp – regulator for lrp regulon and high–affinity branched–chain amino acid transport system
glnG – EBP family response regulator in two–component regulatory system with GlnL
glnK – regulatory protein P–II 2
amtB – putative Amt family, ammonium transport protein
gltD – glutamate synthase, small subunit
gltB – glutamate synthase, large subunit
Effect of M
B
Competitive
sodA – superoxide dismutase
dcuR – response regulator in two–component regulatory system with DcuS
yheO – putative regulator
yohJ – putative effector of murein hydrolase LrgA
yhdH – putative oxidoreductase
maeB – putative transferase
yohK – putative transmembrane protein
dctA – DAACS family C4–dicarboxylic acids transport protein
prkB – putative phosphoribulokinase
dcuS – sensory histidine kinase in two–component retgulatory system with DcuR
yiiD – putative acetyltransferase
maeA – NAD–linked malate dehydrogenase
fbp – fructose–bisphosphatase
cafA – RNase G
cra – transcriptional repressor of fru operon and others
tig – peptidyl–prolyl cis/trans isomerase
srlR – transcriptional repressor for glucitol operon
tatC – component of sec–independent protein export
bioB – biotin synthetase
fadE – putative acyl–CoA dehydrogenase
pckA – phosphoenolpyruvate carboxykinase
flgE – hook protein
yebA – putative peptidase
btuB – outer membrane receptor for transport of vitamin B12, E colicins, and bacteriophage BF23
acrB – RND family, acridine efflux pump
gsiA – putative ATPase components of ABC–type transport system, containing duplicated ATPase domain
rbsK – ribokinase
rbsR – transcriptional repressor for rbs operon (GalR/LacI family)
queA – S–adenosylmethionine–tRNA ribosyltransferase–isomerase
lsrF – putative fructose–1,6–bisphosphate aldolase
talA – transaldolase A
panE – ketopantoate reductase
mntR – putative Mn–dependent transcriptional regulator
metC – cystathionine beta–lyase
gcvP – glycine cleavage complex protein P
yobF – putative cytoplasmic protein
lrp – regulator for lrp regulon and high–affinity branched–chain amino acid transport system
gcvT – glycine cleavage complex protein T
gcvH – glycine cleavage complex protein H
prmC – putative protoporphyrinogen oxidase
purN – polyphosphate kinase
metAS – homoserine transsuccinylase
nadA – quinolinate synthetase, A protein
nadC – quinolinate phosphoribosyltransferase
thrA – aspartokinase I
pdxA – NAD–dependent dehydrogenase/carboxylase
apbC – putative ATP–binding protein
argD – acetylornithine transaminase
panB – 3–methyl–2–oxobutanoate hydroxymethyltransferase
gpmB – putative phosphoglyceromutase 2
glnL – sensory kinase (phosphatase) in two–component regulatory system with GlnG (nitrogen regulator II, NRII)
pabB – p–aminobenzoate synthetase, component I
nadB – quinolinate synthetase, B protein
acs – acetyl–CoA synthetase
yafV – putative amidohydrolase
manA – mannose–6–phosphate isomerase
fldB – flavodoxin 2
trpC – N–(5–phosphoribosyl)anthranilate isomerase
yggS – putative enzyme with a TIM–barrel fold
pdxB – erythronate–4–phosphate dehydrogenase
icc – cyclic 3',5'–adenosine monophosphate phosphodiesterase
sspA – stringent starvation protein A
ilvA – threonine deaminase
ilvE – branched–chain amino–acid aminotransferase
trpE – anthranilate synthase, component I
pdxA2 – pyridoxine phosphate biosynthetic protein
thrB – homoserine kinase
Effect of E
Estimate
6
3
0
−3
−6

Supplementary Figure S5 – Genes with significant fitness effects heatmap. Genes with significant effects (adj. *p* value < 0.05) in the presence of E or M in mutualistic **(A)** and competitive **(B)** conditions. Color indicates the linear regression estimate – fitness improvements (relative to monoculture) are indicated in blue, while decreased fitnesses are indicated in red. Note that no genes were significant for the competition in the presence of M.

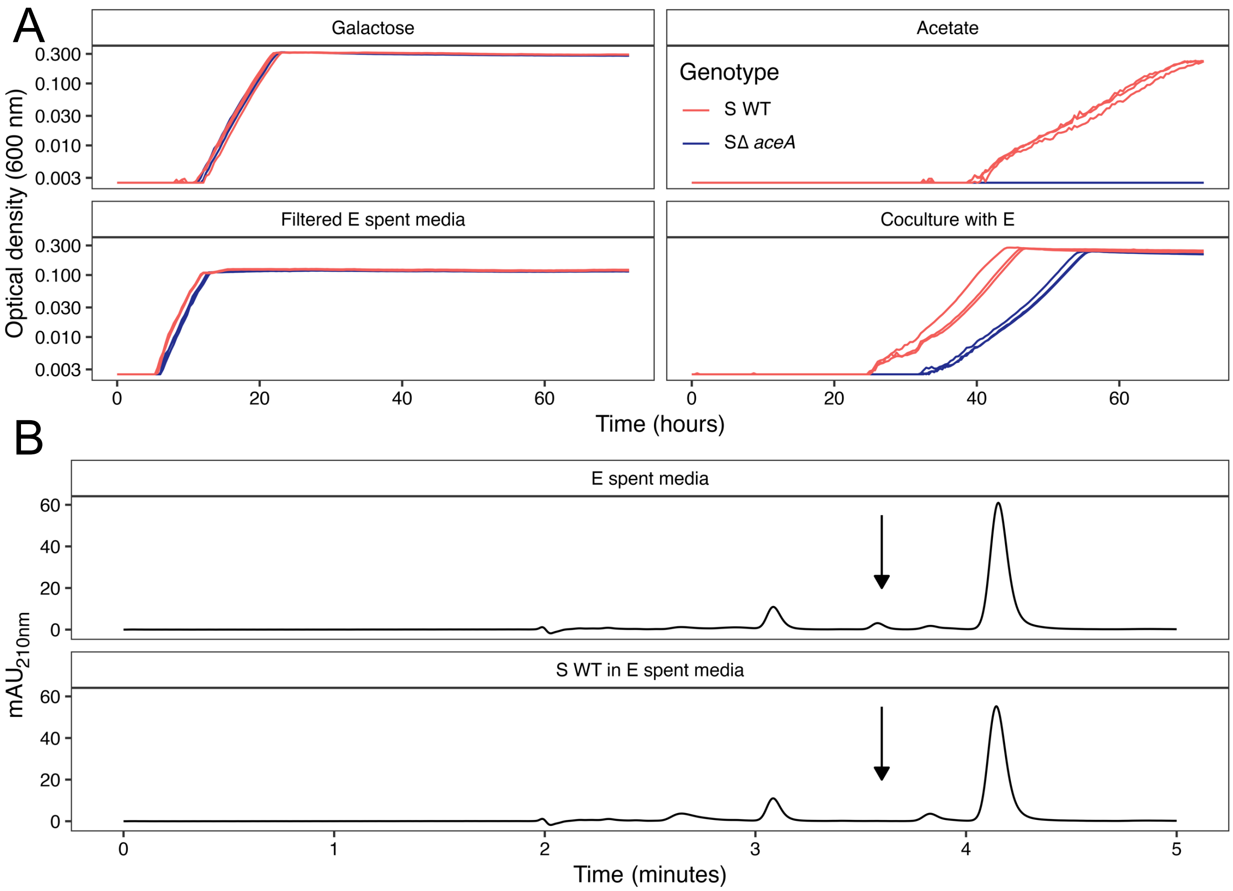

Supplementary Figure S6 – Unexpected carbon catabolism fitness patterns. A Growth curves (OD_{600}) of wild type (red) and Δ*aceA* mutant *S. enterica* (blue) grown in minimal media containing galactose (top left) and acetate (top right) as the sole carbon source. In the bottom left panel growth of the two strains in filter sterilized spent media prepared from mid-log *E. coli* (lactose + methionine minimal media). In the bottom right panel, growth in mutualistic co-culture with *E. coli* in lactose minimal media. The y-axis is $\log_{10}$ transformed. Three replicates are shown in each case. **B** HPLC chromatograms for *E. coli* spent media (top) and *S*. *enterica* grown on *E. coli* spent media (bottom). Standards were used to optimize this method to measure acetate, butyrate, citrate, formate, lactate, propionate, pyruvate, and succinate. The arrow indicates the approximate retention time that acetate elutes (3.6 minutes).

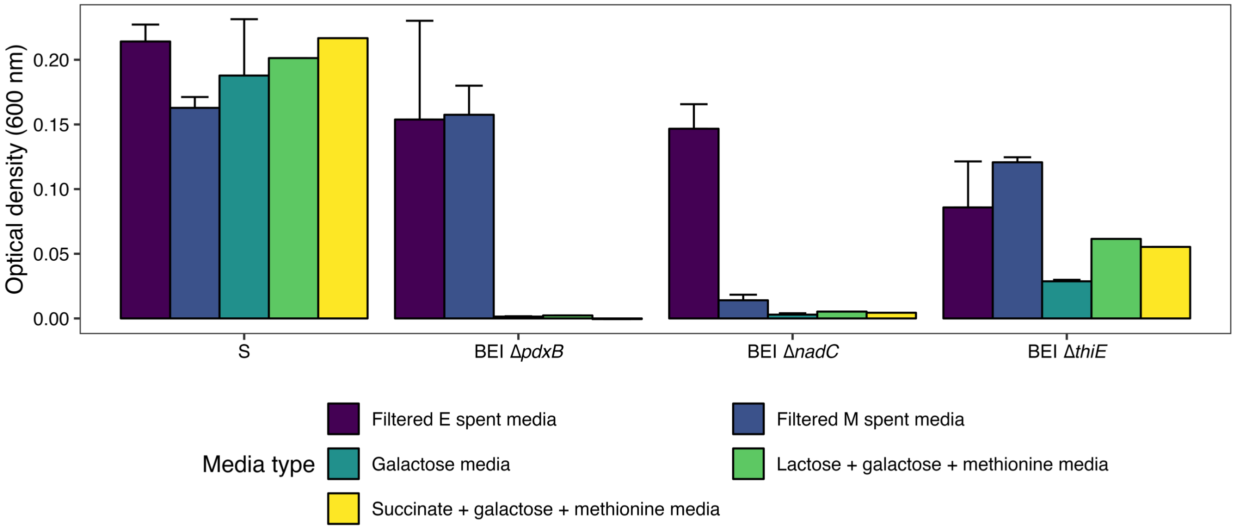

Supplementary Figure S7 – Vitamin and co-factor mutants largely recapitulate BarSeq data. Final OD_{600} of the wildtype *S. enterica* methionine hypersecreter and vitamin/cofactor auxotrophs (Δ*pdxB*, Δ*nadC*, Δ*thiE*) from the BEI knockout collection. Strains were grown in either fresh Hypho minimal media containing various carbon/amino acid sources and spent media prepared from stationary phase *E. coli* or *M. extorquens*. All experiments were conducted in triplicate.

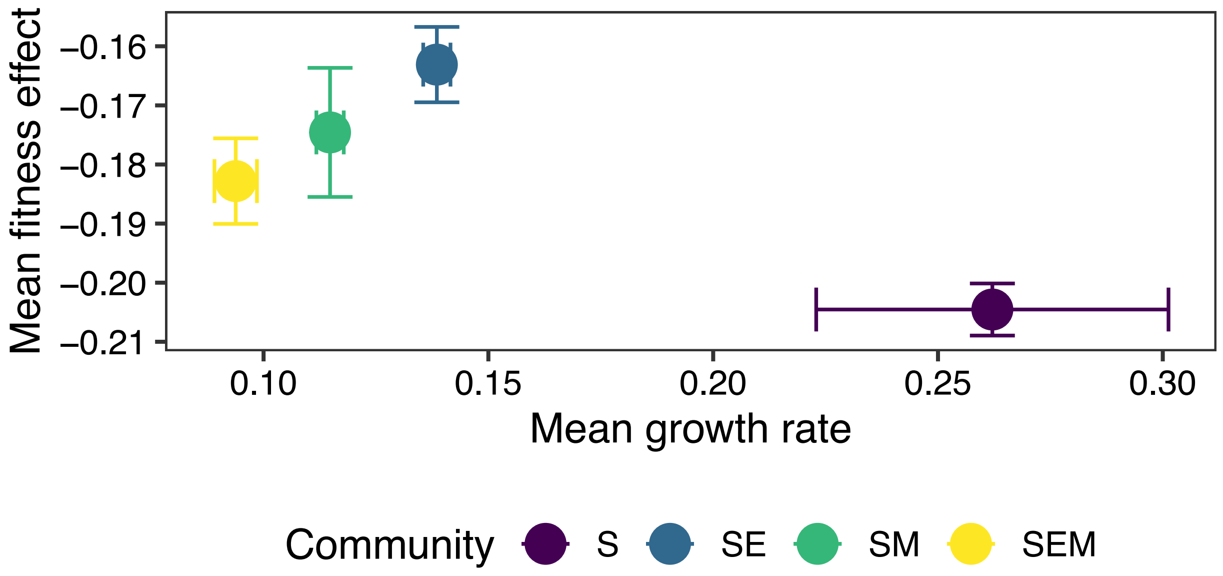

Supplementary Figure S8 – Relationship between growth rate and mean fitness effect in mutualistic treatments. The mean growth rate calculated using the automated scanner method (n = 3) plotted against the mean fitness effect derived from the BarSeq experiment (n = 5) for each community under mutualistic conditions. The error bars represent the standard deviation.
